# Evaluation of Surface Roughness, Hardness, and Gloss of Composites After Three Different Finishing and Polishing Techniques: An In Vitro Study

**DOI:** 10.7759/cureus.7037

**Published:** 2020-02-19

**Authors:** Kumar Nithya, Krishnamoorthy Sridevi, Venkatesan Keerthi, Periasamy Ravishankar

**Affiliations:** 1 Conservative Dentistry and Endodontics, Thai Moogambigai Dental College and Hospital, Dr. M.G.R. Educational and Research Institute University, Chennai, IND

**Keywords:** resin composites, finishing/polishing systems, surface roughness, microhardness, gloss

## Abstract

Introduction

The purpose of this in vitro study was to evaluate the effect of three different polishing systems on the microhardness, surface roughness, and gloss of resin composites.

Materials and Methods

The materials evaluated were 3M™ ESPE™ Filtek™ Z-350 XT (3M™, St. Paul, MN, USA), Grandio (Voco, Cuxhaven, Germany), 3M™ ESPE™ Filtek™ Z250 (3M™, St. Paul, MN, USA), Shofu-Beautifil Flow (Shofu, Kyoto, Japan), and RestoFill HV N-FLO (Anabond Stedman, Chennai, India). A total of 450 samples were fabricated. Three finishing and polishing systems: PoGo® (Dentsply Caulk, Milford, DE, USA), Sof-Lex Spiral, and Sof-Lex Pop-On (3M™, St Paul, MN, USA) were evaluated. Hardness, roughness, and gloss were evaluated after finishing and polishing. The surface roughness was measured with a surface profilometer, microhardness was measured with the Struers Duramin-5 microhardness tester (Struers A/S, Ballerup, Denmark) and gloss was measured using a gloss meter. The measurement values were analysed by Kolmogorov-Smirnov, Shapiro-Wilks test, and two-way ANOVA.

Results

The Sof-Lex Spiral group exhibited higher mean microhardness (p < 0.001), less surface roughness (p < 0.001), and higher gloss (p < 0.001). Filtek Z-250 exhibited higher mean microhardness (p < 0.001) than Grandio (p < 0.001) and Shofu Beautifil Flow (p < 0.001), and Filtek Z-350 XT exhibited more microhardness than Shofu Beautifil Flow (p < 0.001). Filtek Z-350 XT exhibited lower mean surface roughness than Filtek Z-250 (p < 0.05). Filtek Z-250 polished with Sof-Lex Spiral proved to have higher gloss (34.89 gloss units (GU)) than Grandio and RestoFill HV N-FLO (p < 0.05).

Conclusions

Hardest, smoothest, and glossiest surfaces were obtained with the Sof-Lex Spiral finishing/polishing system. The mean microhardness of Filtek Z-250 and Filtek Z-350 XT was found to be higher than other composites used in this study. Filtek Z-350 showed a lower mean surface roughness and Filtek Z-250 exhibited a higher mean gloss.

## Introduction

Aesthetic concepts and mechanical properties have played an important role in the further development of newer dental restorative materials [1-2]. A smooth surface improves the longevity of restoration by reducing plaque retention, gingival inflammation, and recurrent caries [3-4]. Thus, proper techniques for finishing and polishing play a significant role in improving the appearance and lifetime of restorations [5]. The filler particle size, hardness, and distribution in the composite, along with the abrasive agents used for finishing and polishing will determine the final surface characteristics of restorations [6].

Recently, nanocomposites were reported to be superior to hybrid and micro-filled composites as they have esthetic and mechanical properties required for anterior and posterior restorations [7-8]. Hardness is the property of a solid surface to resist indentations [5]. Resin composite microhardness depends on various factors, such as the composition of the organic matrix, along with the type and shape of filler particles [9]. Surface hardness in resin composites is directly related to filler particle concentrations [10].

Surface roughness is dependent on the composition of resin composite and polishing instruments/procedures [11]. The size of filler particles in resin composites has been reported to be an important aspect affecting the transmittance and reflectance of the final restoration [12]. Optical properties, which include color, gloss, and surface texture of composites, are affected by the surface finish achieved during finishing and polishing procedures [13]. Thus, the composition of resin composites and the finishing/polishing system play an important role in influencing surface gloss, roughness, and microhardness [1].

Currently, there is no consensus in the literature on the finishing and polishing instruments recommended for each type of composite [14]. Therefore, the present study investigated the effects of three polishing systems (one-step, two-step, and three-step systems) on the surface hardness, roughness, and gloss of one nanofiller packable, one nanohybrid packable, one micro-hybrid packable, and two nanohybrid flowable resin composites using a profilometer, Vickers hardness test, and gloss meter. The null hypothesis tested was that there was no difference between three finishing and polishing protocols in terms of microhardness, surface roughness, and gloss in the five resin composites evaluated.

## Materials and methods

One nanofiller packable composite (Filtek Z-350 XT (3M™, St. Paul, MN, USA)) with zirconia and silica clusters of 0.6 - 1.4 µm [15], one nanohybrid packable composite (Grandio (Voco, Cuxhaven, Germany)) with 20 - 60 nm glass and silica fillers [4], two nanohybrid flowable composites (Shofu Beautifil Flow (Shofu, Kyoto, Japan) with glass filler particle size -0.01 - 4.0 µm) and RestoFill HV N-FLO (Anabond Stedman, Chennai, India) with fumed silica (60 - 250 nm) and one micro-hybrid packable composite (Filtek Z-250 (3M™, St. Paul, MN, USA)) with 0.01 - 3.5 µm zirconia-silica] with shade A2 and three finishing and polishing (F/P) systems: PoGo® (Dentsply Caulk, Milford, DE, USA), Sof-Lex Spiral (3M™, St. Paul, MN, USA), and Sof-Lex Pop-On (3M™, St. Paul, MN, USA) were evaluated in the present study. PoGo is a one-step polishing system with polymerized urethane dimethacrylate (UDMA) resin, fine diamond powder, and 20 µm silicon oxide [16]. Sof-Lex Spiral is a two-step polishing system composed of elastomer impregnated with aluminum oxide particles (25 - 29 µm) [1]. Sof-Lex Pop-On is a three-step polishing system composed of medium (20 µm), fine (18 µm), and super-fine (14 µm) aluminum oxide-impregnated discs [17].

Preparation of test samples

Three hundred samples were prepared using a cylindrical mold (8 mm in diameter and 2 mm in height) and were evaluated for microhardness, surface roughness, and color. One hundred and fifty samples were prepared using a cylindrical mold (15 mm diameter and 1 mm height) and evaluated for gloss [1]. Each mold was ﬁlled with composite resin and excess material was removed by compressing between two glass slides to obtain a flat surface. The glass slides were later removed and the samples covered by a polyester matrix were polymerized using a light-emitting diode (LED) curing light (LED Elipar Free Light) (3M™, St. Paul, MN, USA) of 1,000 mW/cm^2^ strength and light-cured for 40 seconds. In total, 450 resin discs were prepared for three F/P systems with 90 discs from each resin composite. Later, all discs were stored in distilled water at 37º C for 24 hours prior to testing.

Next, the top surfaces of the discs were ground with 600 grit silicon carbide (SiC) paper for 20 seconds under running water for standardization. Sample preparation and associated F/P procedures were performed according to manufacturer’s instructions using three F/P systems by the same operator to avoid bias.

Microhardness measurements

For the microhardness test, 10 disc-shaped specimens (n = 10) were evaluated for each resin and F/P system. The Vickers hardness number (VHN) was determined using a Struers Duramin-5 microhardness tester (Struers A/S, Ballerup, Denmark). Three indentations were made on the surface under a 200-gram load with a 15 seconds dwell time and the mean was calculated.

Surface roughness measurements

Ten disc-shaped specimens were evaluated for each resin composite and F/P system. The surface roughness (Ra) value was recorded using a two-dimensional profilometer (Surtronic 3+, Taylor Hobson, Leicester, UK) having a 5 µm diamond stylus and an angle of 90° traversing a length of 1.25 mm with a cut-off length of 0.25 mm. Three measurements were performed in the centre of each sample in different directions and the mean was calculated.

Gloss measurements

Gloss measurements expressed in gloss units (GU), were also performed using a gloss meter (GM 26 Glossmeter, Dalian Teren Industry Instrument Co., Ltd., Liaoning, China) with a square measurement area of 15 × 10 mm and a 60° geometry to determine the gloss values of the samples. The gloss meter measures the intensity of a reflected light beam after striking the surface and compares the measured value to a reference value. An opaque black plastic mold was placed over the specimen during measurement to eliminate the influence of ambient light and to maintain the exact position of the sample for repeated measurements. Three measurements were performed for each specimen and the mean was calculated.

Statistical analysis

Kolmogorov-Smirnov and Shapiro-Wilks test results revealed that all variables followed a normal distribution. Therefore, to analyze the data, parametric methods were applied. Two-way ANOVA (general linear model) was used to compare mean values between groups and materials followed by Bonferroni post hoc tests for multiple pairwise comparisons. To analyze the data, the Statistical Package for Social Sciences (SPSS) software, version 23.0 (IBM SPSS Statistics for Windows, Armonk, NY) was used. Significance level was set at 5% (α = 0.05). 

## Results

Surface microhardness

There was a significant difference (p < 0.001) in mean hardness between materials and between groups (p < 0.001). The mean microhardness (VHN) values and standard deviations for the composite resins tested under the experimental conditions used in this study are shown in Table 1.

**Table 1:** Mean Microhardness Values (VHN kg/mm2) of the Tested Resin Composite Materials and Polishing Techniques HV: high viscosity

Material	Group	Mean	Standard Deviation	N
Grandio	PoGo	92.9	6.67	10
	Sof-Lex Spiral	106.4	8.80	10
	Sof-Lex Pop-On	85.5	8.77	10
	Total	94.9	11.81	30
Filtek Z-350	PoGo	91.0	8.78	10
	Sof-Lex Spiral	104.7	5.63	10
	Sof-Lex Pop-On	100.3	6.21	10
	Total	98.6	8.91	30
Filtek Z-250	PoGo	98.4	6.98	10
	Sof-Lex Spiral	104.8	7.53	10
	Sof-Lex Pop-On	97.5	4.61	10
	Total	100.3	7.09	30
Shofu Beautifil Flo	PoGo	91.8	5.39	10
	Sof-Lex Spiral	91.5	6.11	10
	Sof-Lex Pop-On	87.2	6.26	10
	Total	90.2	6.12	30
RestoFill HV N-FLO	PoGo	81.7	5.74	10
	Sof-Lex Spiral	79.3	8.76	10
	Sof-Lex Pop-On	78.5	7.37	10
	Total	79.8	7.27	30
Total	PoGo	91.1	8.51	50
	Sof-Lex Spiral	97.4	12.81	50
	Sof-Lex Pop-On	89.8	10.39	50
	Total	92.8	11.14	150

Sof-Lex Spiral group had significantly more microhardness than PoGo group (p < 0.001) and Sof-Lex Pop-On group (p < 0.001). Filtek Z-250 had significantly more microhardness than Grandio (p < 0.001) and Shofu Beautifil Flow (p < 0.001). Filtek Z-350 XT had significantly more microhardness than Shofu Beautifil Flow (p < 0.001). RestoFill HV N-FLO had significantly less microhardness (p < 0.001) than all other materials. All other paired comparison between materials were statistically not significant.

Surface roughness

The mean surface roughness (Ra, µm) values and standard deviations for the composite resins tested under the experimental conditions used in this study are shown in Table 2.

**Table 2:** Mean Values and Standard Deviations of Surface Roughness (Ra, µm) of Resin Composites and Polishing Techniques HV: high viscosity

Material	Group	Mean	Standard Deviation	N
Grandio	PoGo	0.676	0.252	10
	Sof-Lex Spiral	0.421	0.111	10
	Sof-Lex Pop-On	0.713	0.092	10
	Total	0.603	0.209	30
Filtek Z-350	PoGo	0.657	0.146	10
	Sof-Lex Spiral	0.420	0.104	10
	Sof-Lex Pop-On	0.660	0.152	10
	Total	0.579	0.174	30
Filtek Z-250	PoGo	0.821	0.105	10
	Sof-Lex Spiral	0.493	0.083	10
	Sof-Lex Pop-On	0.698	0.043	10
	Total	0.670	0.158	30
Shofu Beautifil Flo	PoGo	0.706	0.132	10
	Sof-Lex Spiral	0.499	0.134	10
	Sof-Lex Pop-On	0.736	0.103	10
	Total	0.647	0.161	30
RestoFill HV N-FLO	PoGo	0.645	0.114	10
	Sof-Lex Spiral	0.580	0.070	10
	Sof-Lex Pop-On	0.696	0.080	10
	Total	0.640	0.099	30
Total	PoGo	0.701	0.165	50
	Sof-Lex Spiral	0.482	0.115	50
	Sof-Lex Pop-On	0.700	0.099	50
	Total	0.628	0.165	150

Filtek Z-250 finished with PoGo F/P system showed the highest mean roughness of 0.82 µm. The Sof-Lex Spiral group had significantly less roughness than the PoGo group (p < 0.001) and the Sof-Lex Pop-On group (p < 0.001). Filtek Z-350 XT had significantly less roughness (p < 0.05) than Filtek Z-250. All other paired comparisons between materials were not statistically significant (p > 0.05). In the Sof-Lex Spiral group, Filtek Z-350 XT had significantly less roughness than Restofill HV N-FLO and Grandio (p < 0.05).

Gloss

The mean gloss values (GU) and standard deviations for the resin composites used in this study are shown in Table 3.

**Table 3:** Mean Gloss Values (GU) and Standard Deviation (± SD) for the Composites and Polishing Systems Evaluated HV: high viscosity

Material	Group	Mean	Standard Deviation	N
Grandio	PoGo	22.63	3.13	10
	Sof-Lex Spiral	30.68	5.09	10
	Sof-Lex Pop-On	25.65	4.15	10
	Total	26.32	5.28	30
Filtek Z-350	PoGo	26.82	5.26	10
	Sof-Lex Spiral	33.43	5.02	10
	Sof-Lex Pop-On	28.37	5.07	10
	Total	29.54	5.71	30
Filtek Z-250	PoGo	28.58	3.90	10
	Sof-Lex Spiral	34.89	4.41	10
	Sof-Lex Pop-On	28.98	4.67	10
	Total	30.82	5.11	30
Shofu Beautifil Flo	PoGo	26.78	5.18	10
	Sof-Lex Spiral	32.88	6.51	10
	Sof-Lex Pop-On	28.04	3.18	10
	Total	29.23	5.64	30
RestoFill HV N-FLO	PoGo	25.42	4.48	10
	Sof-Lex Spiral	27.03	4.81	10
	Sof-Lex Pop-On	26.89	4.08	10
	Total	26.45	4.37	30
Total	PoGo	26.05	4.72	50
	Sof-Lex Spiral	31.78	5.71	50
	Sof-Lex Pop-On	27.59	4.27	50
	Total	28.47	5.47	150

The highest gloss was exhibited by all composites polished with Sof-Lex Spiral (p < 0.001). Filtek Z-250 had a significantly higher mean gloss than Grandio (p < 0.05) and RestoFill HV N-FLO (p < 0.05). 

## Discussion

Resin composites have been widely used in recent times due to increasing esthetic demands by patients and technological advancements in the field of dentistry [18]. Surface smoothness and gloss are two characteristics comparable to natural enamel and should be replicated to achieve natural tooth form and esthetics [18-19]. The handling characteristics and aesthetic properties of resin composites are usually affected by the type of fillers and filler content, but the final outcome of the restoration is strongly influenced by the finishing and polishing techniques [1, 20-21]. Hence, this in vitro analysis was done to evaluate the effects of three different F/P protocols on the hardness, surface roughness, and gloss of different resin composites. In the present study, Filtek Z-250 had significantly higher mean microhardness than Grandio and Shofu Beautifil Flow, while RestoFill HV N-FLO exhibited significantly lower mean microhardness values (p < 0.001). Increased filler levels can result in increased surface hardness [11, 22], compressive strength and flexural strength [22]. Similarly, RestoFill HV N-FLO with 60% filler by weight had the least mean microhardness.

The highest mean Ra value for all composite materials tested in the current study was 0.82 μm which was produced by the Filtek Z-250 and PoGo F/P systems. It has been reported that restorations with a Ra value of less than 1 µm appear to be optically smooth [23]. Therefore, all resin composites used in this study produced optically acceptable Ra values with the polishing systems tested. In the present study, Sof-Lex Spiral created significantly smoother surfaces than Sof-Lex Pop-On and PoGo F/P systems for all resin composites. The flexible wheel design can adapt to most surfaces of a restoration resulting in improved polish [1, 24]. In accordance with our results, Sof-Lex Spiral has been reported as an effective instrument for producing smooth surfaces due to its ability to remove both organic matrix and filler particles [25]. Surface roughness values of the Filtek Z-350 XT polished with the Sof-Lex Spiral group were significantly lower than the Filtek Z-250 polished with Sof-Lex Spiral. All materials polished with Sof-Lex Spiral had significantly more gloss than materials polished with PoGo or Sof-Lex Pop-On. Similar results have been reported suggesting that multistep finishing and polishing systems produced higher gloss than one-step finishing and polishing system [15]. Filtek Z-250 exhibited higher mean gloss than Grandio and RestoFill HV N-FLO. According to the American Dental Association (ADA) professional product review, restorations with typically desired gloss exhibited 40 - 60 GU [26]. According to the present study, none of the composite resin materials exhibited the desired gloss results with gloss values between 22.6 and 34.08 GU. The irregular-shaped particles in micro-hybrid and nano-hybrid resin composites used in the present study may impair the production of a smooth, reflective surface when compared to round-shaped filler particles [27]. When surface roughness is increased, decreased gloss occurs [28]. Results of our study showed that resin composites polished with Sof-Lex Spiral had lower surface roughness and higher gloss compared with other F/P systems. F/P procedures, as well as aging, can affect the physicomechanical properties and longevity of restorations [1]. One of the limitations of the present study is that composite resin samples were not evaluated after thermocycling. Within the limitations of the present study, we conclude that there is a significant difference between the groups. Hence, the null hypothesis is rejected, suggesting that there is a significant difference between three F/P systems in terms of microhardness, surface roughness, and gloss.

## Conclusions

Within the limitations of this study, composites polished with the Sof-Lex Spiral system exhibited more microhardness, less surface roughness, and higher gloss. Filtek Z-250 and Filtek Z-350 XT showed higher microhardness values. The maximum smoothness and glossiness were achieved with Filtek Z-350 XT and Filtek Z-250 composites, respectively.
